# Machine-Learning Points at Endoscopic, Quality of Life, and Olfactory Parameters as Outcome Criteria for Endoscopic Paranasal Sinus Surgery in Chronic Rhinosinusitis

**DOI:** 10.3390/jcm10184245

**Published:** 2021-09-18

**Authors:** Jörn Lötsch, Constantin A. Hintschich, Petros Petridis, Jürgen Pade, Thomas Hummel

**Affiliations:** 1 Institute of Clinical Pharmacology, Goethe-University, Theodor-Stern-Kai 7, 60590 Frankfurt am Main, Germany; 2 Fraunhofer Institute for Translational Medicine and Pharmacology ITMP, Theodor-Stern-Kai 7, 60596 Frankfurt am Main, Germany; 3 Department of Otorhinolaryngology, University of Regensburg, Franz-Josef-Strauß-Allee 11, 93053 Regensburg, Germany; constantin.hintschich@klinik.uni-regensburg.de; 4 Smell & Taste Clinic, Department of Otorhinolaryngology, TU Dresden, Fetscherstrasse 74, 01307 Dresden, Germany; thummel@mail.zih.tu-dresden.de; 5 Department of Otorhinolaryngology, St. Johannes Municipal Hospital, Johannesstraße 9-17, 44137 Dortmund, Germany; petridispppeter@hotmail.com (P.P.); juergen.pade@gmx.de (J.P.)

**Keywords:** otorhinolaryngology, human olfaction, machine learning, data science, patients

## Abstract

Chronic rhinosinusitis (CRS) is often treated by functional endoscopic paranasal sinus surgery, which improves endoscopic parameters and quality of life, while olfactory function was suggested as a further criterion of treatment success. In a prospective cohort study, 37 parameters from four categories were recorded from 60 men and 98 women before and four months after endoscopic sinus surgery, including endoscopic measures of nasal anatomy/pathology, assessments of olfactory function, quality of life, and socio-demographic or concomitant conditions. Parameters containing relevant information about changes associated with surgery were examined using unsupervised and supervised methods, including machine-learning techniques for feature selection. The analyzed cohort included 52 men and 38 women. Changes in the endoscopic Lildholdt score allowed separation of baseline from postoperative data with a cross-validated accuracy of 85%. Further relevant information included primary nasal symptoms from SNOT-20 assessments, and self-assessments of olfactory function. Overall improvement in these relevant parameters was observed in 95% of patients. A ranked list of criteria was developed as a proposal to assess the outcome of functional endoscopic sinus surgery in CRS patients with nasal polyposis. Three different facets were captured, including the Lildholdt score as an endoscopic measure and, in addition, disease-specific quality of life and subjectively perceived olfactory function.

## 1. Introduction

Chronic rhinosinusitis (CRS) has an estimated prevalence in the European population ranging from 6.9 to 27.1% [1]. In addition to nasal discharge, congestion, and facial pain/pressure, its clinical symptoms often include olfactory dysfunction [2,3]. Two mechanisms have been identified that contribute to the olfactory symptoms. First, olfactory loss in the CRS subtype with nasal polyps (CRSwNP) has mechanical causes, as polyps, mucosal edema, or pus can obstruct the olfactory cleft and impede access of odor molecules to the olfactory epithelium [4]. Second, in both subtypes of CRS, i.e., CRSwNP and CRS without nasal polyps (CRSsNP), inflammatory processes can extend to the olfactory epithelium where they cause local sensorineural damage [5,6].

First-line therapies for CRS include nasal irrigation and topical steroids [7]. When this fails, functional endoscopic nasal sinus surgery is the common treatment for persistent CRS, especially in CRSwNP, which has been shown to significantly improve specific symptoms as well as quality of life [8]. It has also been shown to recover olfactory performance [9,10], but it has remained unclear whether this is a concomitant benefit or a relevant inherent component contributing to the improved quality of life reported after surgery [11]. Its repeatedly demonstrated impact on quality of life under different circumstances [12,13,14] does not automatically make olfactory function a criterion by which to evaluate the success of surgical treatment of CRS. A role of olfactory function was previously indicated in an analysis of olfactory and quality of life related parameters collected in a sinus surgery context [15]. However, the regression analysis provided merely statistical significances of olfactory measures without ranking them clearly among different measures such as endoscopic scores.

The present study aimed at criteria that characterize the success of surgical treatment of CRS in a relevant way. Several parameters each (two or more tests) from a total of four main categories related to (i) nasal anatomy/pathology, (ii) olfactory function, (iii) quality of life, and (iv) demographic data or relevant concomitant diseases were recorded. To obtain robust cross-validated results, a data-driven biomedical informatics-based approach [16] was used to select for each category those parameters that carry most relevant information that can be used to identify whether a set of parameters has been recorded either before or after endoscopic sinus surgery. The present approach used machine learning in conjunction with so-called feature selection methods, i.e., the selection of a subset of relevant variables for the operation of the algorithms [17].

## 2. Materials and Methods

### 2.1. Study Design and Participants

Adopting a prospective open design, this observational cohort study took place at two consecutive occasions, before the surgery and four months postoperatively. The cohort included 158 patients with nasal polyps, i.e., 60 men and 98 women, aged 13.9–84.6 years (mean ± standard deviation: 49.1 ± 14.8 years), who were preparing for endoscopic sinus surgery at the Department of Otorhinolaryngology, St. Johannes Municipal Hospital, Dortmund, Germany. Measurements took place between May 2018 and August 2019. Patients were diagnosed according to the EPOS guideline [18]. Inclusion criteria were absence of pregnancy, absence of disorders that are strongly associated with olfactory loss such including neurodegenerative disorders such as Parkinson’s or Alzheimer’s disease, or advanced renal dysfunction. The study was conducted in accordance with the Declaration of Helsinki on Biomedical Studies Involving Human Subjects. It was approved by the Ethics Committee at the Dresden University Hospital (approval number EK14502017). All participants gave informed written consent and in the case of minors their parents’ informed written consent was additionally obtained. The sample size was defined to be twice that of a positive study of the correlation of measured olfactory function with self-assessments of olfaction in *n* = 80 patients with nasal polyposis [19]. A formal sample size estimate was not performed.

### 2.2. Data Acquisition

Data collected included (i) endoscopic measurements of nasal anatomy/pathology, (ii) assessments of olfactory function, (iii) quality of life questionnaire responses, and (iv) sociodemographic and concomitant disease-related information. The exact methods used to collect this information are described below.

#### 2.2.1. Assessment of Nasal Anatomy/Pathology

##### Visual Scoring of Pathological Conditions in the Nose and Paranasal Sinuses

Endoscopic examination of the nasal cavity was performed by two experienced otolaryngologists (PP, JP). **First**, the presence and severity of potential polyps, edema, discharge, scarring, and crusting were each scored between 0 and 2, assessed for each side, and summed to the Lund–Kennedy score [20]. No significant findings on endoscopic evaluation are concluded from a Lund–Kennedy score of <2 [21]. **Second**, polyp size was evaluated separately for each side and then summed to the Lildholdt score [22]. The coding was 0, no polyps; 1, polyps not reaching the inferior turbinate; 2, polyps not reaching the inferior edge of the inferior turbinate; 3, polyps reaching below the inferior edge of the inferior turbinate.

##### Assessment of Eosinophilia in Nasal Tissue

The presence or absence of eosinophilia was determined in the histologic specimen by a trained clinical pathologist. Tissue (polyps, mucosa) collected during nasal surgery was examined histologically in all patients similar to the methods of Bachert and Holtappels [23]. Eosinophilia in the tissue is indicative of a Th2 inflammatory pattern associated with frequent recurrences of polyposis and asthma [23,24].

#### 2.2.2. Assessment of Olfactory Function

##### Clinical Testing of Olfactory Functional Performance

Olfactory function was quantified using a validated and reliable clinical test (“Sniffin’ Sticks”, Burghart Instruments, Wedel, Germany) [25,26], which evaluated three sensory dimensions of odors comprising olfactory threshold (to phenylethyl alcohol), odor discrimination (16 pairs of odors), and odor identification (16 odors). From the sum score, the olfactory functional diagnosis was obtained as either functional anosmia (further termed “anosmia”; score < 16.5), hyposmia, (16.5–30.5), or normosmia (>30.5) [27]. Regardless of this olfactory diagnostic classification, an improvement in olfactory function is subjectively perceived as such when the TDI increases by ≥5.5 points [28].

##### Self-Ratings of the Perceived Olfactory Function

Participants rated their olfactory function in two ways on two different Likert-type scales. **Rating scale #1** was an 8-point scale, with each point being labeled as 0 = “no smell perception”, 1 = “extremely bad”, 2 = “much worse than normal”, 3 = “worse than normal”, 4 = “normal sense of smell”, 5 = “better than normal”, 6 = “much better than normal”, and 7 = “excellent”. **Rating scale #2** was a discrete scale, with labels only at endpoints of the scale, with 10 data points on which subjects rated their olfactory function from 1, “not present” to 10, “excellent”.

The inclusion of self-assessments of olfactory function has been the subject of intense debate in the planning of the study data analysis, as the scientific value of these queries can be judged differently. On the one hand, self-assessments of olfactory function have been shown to provide only a limited estimate of olfactory function quantified by a validated clinical test [29]. On the other hand, they have been reported to be highly informative in the clinical setting of nasal polyposis [19,30]. As a result of this discussion, baseline ratings and tests of olfactory function were analyzed for 157 subjects, with the separately published finding that olfactory self-ratings proved informative for assigning categorical olfactory diagnosis [31].

##### Further Olfaction-Related Information

In addition to the testing or rating of the general olfactory acuity, the presence or absence of specific symptoms comprising parosmia [32] and phantosmia [33] was queried from the patients with reference to a validated questionnaire [34].

#### 2.2.3. Assessment of the Quality of Life

##### Assessment of Disease-Specific Quality of Life Related Parameters

The SNOT-20 questionnaire [35] was used as a well-established questionnaire to quantify sinonasal symptoms and assess to treatment outcomes in chronic rhinosinusitis. It consists of 20 questions categorized into 5 different domains (rhinologic symptoms, extranasal rhinologic symptoms, ear/face, psychological dysfunction, sleep dysfunction), which are rated on a Likert scale from 0 = “no problem” to 5 = “it can’t get any worse”. In addition to the general sum score, three different subscores are derived from subsets of responses to the SNOT-20 questionnaire to address specific facets of quality of life. This includes the subscore “**general quality of life**” that contains the individual questions about dizziness, problems with waking up at night, fatigue during the day, diminished performance, poor concentration frustration/restlessness/irritability, sadness, and embarrassment of the disease symptoms. It is calculated as $\sum \left(rating\#11,13,\ldots ,20\right)/45\cdot 100$, where the value of 45 accounts for the maximum sum of individual response to nine questions and the numbers #11, … refer to the item numbers of the SNOT-20. The subscore “**primary nasal symptoms**” combines the questions about nasal obstruction, sneezing, constant nasal secretion, thick mucus nasal secretion, and olfactory impairment, which are transferred into the subscore as $\sum \left(rating\#1,2,3,5,10\right)/25\cdot 100$. The subscore “**secondary nasal symptoms**” combines the questions about secretion flowing into the throat, clearing of the throat, cough, feeling of pressure on the ears, ear, and facial pain, and feeling of pressure in the face, which are transferred into the subscore as $\sum \left(rating\#4,6,\ldots ,9,12\right)/30\cdot 100$. No to minor symptoms are inferred from a total sum score < 12 or from general quality of life < 14, primary nasal symptoms < 12, or secondary nasal symptoms < 10.5 [36].

##### Assessment of Non-Disease-Specific Quality of Life Related Parameters

**Figure 1:**
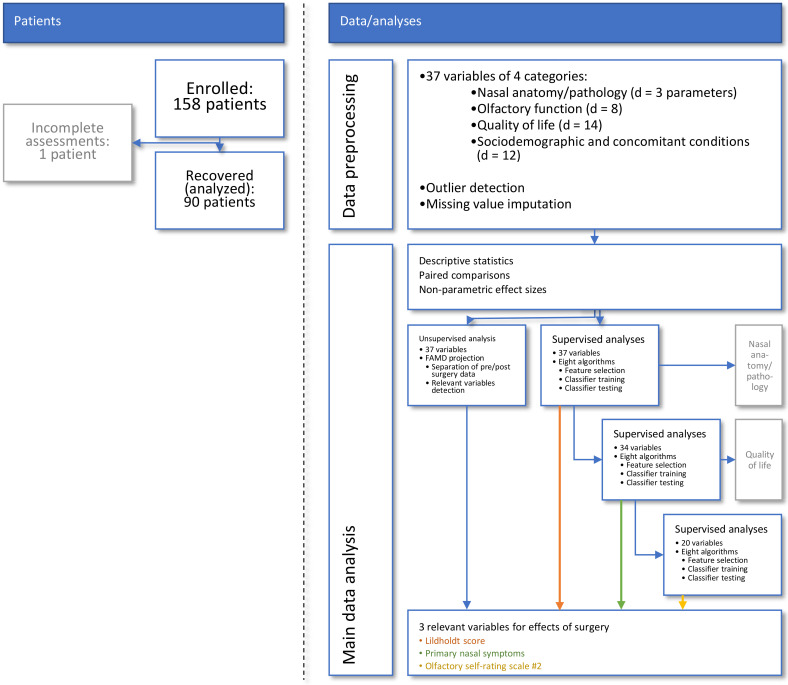
**Flow chart showing the number of patients and data analysis steps.** Major steps in data analysis reached from preprocessing to unsupervised and supervised analyses until variables relevant to capturing the effect of endoscopic endonasal surgery were identified and internally validated. The figure has been created using Microsoft PowerPoint^®^ (Redmond, WA, USA) on Microsoft Windows 11 running in a virtual machine powered by VirtualBox 6.1 (Oracle Corporation, Austin, TX, USA).

In addition, quality of life before and after surgery was surveyed using the German version [37] of the Short Form Survey (SF) 36 [38,39,40] as a widely used self-administered questionnaire for this purpose [41,42]. From 35 questions, an 8-scale profile of physical and mental health measures is calculated by combining responses to selected questions (see, for example, Figure 1 in reference [40]). These subscores cover eight dimensions of health, including (i) physical functioning, (ii) role limitations due to physical health problems, (iii) physical pain, (iv) general health perception, (v) vitality, (vi) social functioning, (vii) role limitations due to emotional problems, and (viii) mental health. In addition, two summary measures addressing (i) physical health (PCS) and (ii) mental health (MCS) are obtained by weighting and averaging the eight subscores. For comparability, U.S. scores were used [43,44]. The SF-36 contains a single further item asking about perceived change in health status, which was queried to fully apply the questionnaire but was not analyzed because its reference point would have been unclear in the baseline survey.

#### 2.2.4. Acquisition of Sociodemographic and Concomitant Disease-Related Information

Sociodemographic parameters included the patient’s age, sex, and body mass index (BMI). Information related to concomitant diseases included (i) the degree of tissue eosinophila, (ii) the triple combination of asthma, pseudoallergy to acetylsalicylic acid and nasal polyps, or pseudoallergy to acetylsalicylic acid or asthma alone, and allergies relevant to nasal or olfactory function. In addition, the use of steroids was recorded as a further binary variable (yes/no).

### 2.3. Data Analysis

A schematic overview of the data analysis workflow can be found in Figure 1. The data analysis was designed to identify from the acquired information those parameters that best captured the changes induced by the endoscopic surgery. Following data preprocessing, the **first** analysis step focused on descriptive and exploratory analyses, such as statistical assessment of significant changes from baseline to postoperative time point and associated effect sizes. **Second**, unsupervised data analyses were performed that used data projection methods to identify structures in the data that support a change from the pre- to the post-surgery state. **Third**, supervised methods including machine-learned classifiers were used to determine the individual parameters that contained relevant information to the changes brought about by the operation. The idea behind this analytical step was to train machine-learning algorithms to identify an individual set of the d = 37 parameters (Table 1) as being recorded from the respective patient before or after surgery and to analyze which specific parameters the algorithms needed to successfully perform this task [45]. By focusing on relevant information about changes caused by surgery, specific parameters should be identified that could serve as criteria to evaluate the outcome of functional endoscopic sinus surgery. This relates to, but is not identical to, analysis of significant changes in selected parameters after surgery. The latter was expected because of the selection of parameters that have been shown to improve after surgery.

The programming work was performed in the R language [46] using the R software package [47], version 4.1 for Linux, which is available free of charge in the Comprehensive R Archive Network (CRAN) at https://CRAN.R-project.org/ (accessed on 16 September 2021). Analyses were performed on 1–26 cores of an Intel Core i9-7940X® (Intel Corporation, Santa Clara, CA, USA) computer with 128 GB of random-access memory (RAM), up to about 60% of which was used during feature selection, running on Ubuntu Linux 20.04.2 LTS (Canonical, London, UK). Parallel processing was programmed using the implementation of the “parallel” R library provided with the R base environment [47].

#### 2.3.1. Data Preprocessing

Preprocessing of the data included (i) examination of the distribution of the variables, possibly followed by data transformation, (ii) detection and removal of outliers, and (iii) imputation of missing values. Different possible transformations along Tukey’s ladder of powers [48] were evaluated and the resulting distributions of the variables compared with the normal distribution using quantile-quantile plots and Kolmogorov–Smirnov tests [49]. This supported logarithmic transformation of olfactory thresholds, which was consistent with the geometric scaling of odorant dilution applied during their acquisition. Outliers were detected by applying Grubbs tests [50] and replaced with missing values. The procedure was iteratively repeated for each variable as long as significant results of Grubbs tests were obtained, using R library “outliers” (https://cran.r-project.org/package=outliers (accessed on 16 September 2021) [51]). Missing values were imputed separately for the four main types of parameters, i.e., nasal-anatomical, olfactory, life quality or sociodemographic and disease-related variables, using median or random forests [52,53] based imputation; the latter implemented in the R library “mice” (https://cran.r-project.org/package=mice (accessed on 16 September 2021) [54]). For further analyses, binary (yes/no) or nominal variables were one-hot recoded with assigning missing values to the zero condition.

#### 2.3.2. Explorative Analyses

Differences between pre- and postoperative values were analyzed using paired tests, which were χ2-statistics [55] or Wilcoxon–Mann–Whitney U tests [56,57]. The α-level was set at 0.05. Because this was an exploratory analysis, no correction for multiple testing was made, but *p*-values are reported accurately so that this can be done at any time.

Effect sizes of the changes associated with the surgery were qualified using the non-parametric effect size measure “Impact” [58] because it is applicable to the different types of variables as analyzed in the present data set, including interval and ordinal scale variables and on-hot transformed nominal variables. As shown previously, in cases where Cohen’s d [59] is defined as a commonly used effect size measure, Impact provides comparable effect sizes [58]. In the present analysis, effect sizes were calculated 1000 times for data sets randomly drawn from the original data set using bootstrap resampling [60]. The 95% confidence intervals (CI) of the effect sizes were determined as the range between the 2.5th and 97.5th percentiles of the respective values during the 1000 runs. These calculations were performed using our R library “ImpactEffectsize” (https://cran.r-project.org/package=ImpactEffectsize (accessed on 16 September 2021) [58]).

#### 2.3.3. Unsupervised Identification of Data Structures Supporting Pre- to Post-Surgery Changes

Whether the data contained a structure that reflected changes from before to after surgery was examined after projecting the high-dimensional 37 × 180 (d × 2*n*) sized data space $D=\left\{x_{i}|x_{i}\in \;X,\;i=1\ldots n\right\}$ onto a low-dimensional plane. Specifically, this data space was composed of the information contained in d = 37 variables collected from the 90 patients at baseline and after surgery who had reported for the two scheduled sessions (for the recovery rate of patients, see the Results section). Variables unchanged between the two acquisitions were included for possible subgroup differences.

Data projection was performed by means of factor analysis for mixed data (FAMD [61]) that combined principal component analysis (PCA [62,63]) for interval (or ordinally) scaled variables with multiple correspondence analysis (MCA [64,65]) as the corresponding technique for binary (categorical) variables in the data set. Variables suitable for the PCA component were scaled to comparable variances and centered according to the R libraries’ defaults. Dimensions with eigenvalues > 1 [66,67] were retained. Analyses of the FAMD projection focused on dimensions on which significant separations were observed between the two tests (before–after) and between the relevant outcomes related to the disease-specific quality-of-life scores identified in the previous step of data analysis, using Wilcoxon–Mann–Whitney U tests [56,57]. The analyses were done using the R library “FactoMineR” (https://cran.r-project.org/package=FactoMineR (accessed on 16 September 2021) [68]). The projection-relevant variables in the focused dimensions were selected as suggested in the R library “factoextra” (https://cran.r-project.org/package=factoextra (accessed on 16 September 2021) [69,70]), which was based on the expected value if the contributions were uniform.

#### 2.3.4. Supervised Identification of Parameters That Carry Relevant Information about Surgery-Related Changes

The above-mentioned data space was completed with the information about the time of acquisition of each data set instance, i.e., baseline or after surgery, at which a respective data set was acquired from a patient. This provided a labeled 37 × 180 ((d + 1) × 2*n*) sized data space $D=\left\{\left(x_{i},y_{i}\right)|x_{i}\in \;X,\;y_{i}\in Y,\;i=1\ldots n\right\}$ that in addition to the composition mentioned with the unsupervised analyses comprising the input space *x_i_* contained an output data space, *y_i_*, that included the test occasion before or after the surgery as a class information.

In this data space, feature selection methods [17] including supervised algorithms from machine learning were used to identify variables that contain relevant information about changes from before to after the surgery; a comparable approach has been published previously [45,71]. That is, different algorithms were trained with 2/3 of the data to identify a data set as acquired either at baseline or post-surgery. The trained algorithms were then put to identify the acquisition time of a data set from the 1/3 of the data not available during training, and the accuracy by which this assignment was performed was quantified. Subsequently, the variables were left out one by one from training, and the decrease in assignment accuracy was kept as a quantitative measure of variable importance, i.e., ${∆}_{Acc}=Acc_{complete}-Acc_{reduced}$, where *Acc_complete_* denotes the accuracy of the test data subset obtained with the complete set of variables, and *Acc_reduced_* denotes the accuracy obtained with one variable omitted. The most relevant variables were then selected by applying an item categorization technique implemented as a computed ABC analysis [72], which divides each set of positive numeric items into three non-overlapping subsets named “A”, “B”, and “C” [73]. Subset “A” contains the “important few” and its member variables were retained. The algorithm was then re-trained with only the retained variables. If it then assigned cases to the correct acquisition time with the same accuracy as the algorithm trained with the full set of variables, it could be concluded that all relevant information had been preserved in the selected variables.

The above workflow was run in a 1000-fold cross-validation scenario as advised for example in [74], using Monte-Carlo [75] resampling to split the data set class-proportionally into two disjoint subsets, of which 2/3 of the original data served as the training data subset and the remaining 1/3 served as the test data subset. The size of the final set of variables (features) selected corresponded to the most frequent size of subsets “A” in the 1000 runs, and its members were the variables most frequently placed in the ABC subset “A” in the 1000 runs, in descending order of their occurrence in the retained sets. These calculations were performed using our R package “ABCanalysis” (https://cran.r-project.org/package=ABCanalysis (accessed on 16 September 2021) [72]) and the R library “sampling” (https://cran.r-project.org/package=sampling (accessed on 16 September 2021) [76]).

Class assignment performance was assessed by calculating standard performance measures [77,78,79,80], balanced accuracy for unequal class sizes [81], and area under the AUC-ROC. These calculations were performed using the R libraries “caret” (https://cran.r-project.org/package=caret (accessed on 16 September 2021) [82]) and “pROC” (https://cran.r-project.org/package=pROC (accessed on 16 September 2021) [83]). The 95% confidence intervals (CI) of the classification performance parameters were determined from the results of the 1000 runs as described above.

A variety of algorithms were used in these analyses, including (i) random forests [52,53] as a generally well-performing ensemble learning classifier that uses a tree-based structure and is implemented in the R library “randomForest” (https://cran.r-project.org/package=randomForest (accessed on 16 September 2021) [84]) and (ii and iii) binary logistic regression as a classical statistical technique routinely used in similar analyses of biomedical datasets and implemented in two different variants of R libraries “nnet” (https://cran.r-project.org/package=nnet (accessed on 16 September 2021) [85]) or the “stats” package from the R base environment. Furthermore, (iv) support vector machines from the R library “e1071” (https://CRAN.R-project.org/package=e1071 (accessed on 16 September 2021) [86]) were used as another usually well-performing classifier. These are based on finding hyperplanes as an optimal decision surface that can separate the data points of one class from those belonging to another class in the high-dimensional feature space [87]. In addition, (v) a k-nearest neighbors (kNN) classifier [88] was used as a prototype based classifier that uses a similarity measure, presently implemented with the Euclidean distance and k = 7 and performed using the R package “KernelKnn” (https://cran.r-project.org/package=KernelKnn (accessed on 16 September 2021) [89]). Finally, (vi) a naïve Bayesian classifier was used as a probabilistic classifier based on the Bayes theorem [90]. These calculations were performed using the R library “e1071”. This selection of classifiers provided a heterogeneous set of algorithms to ensure that the results were not due to properties or implementations of a given type of classifier or its potential problems with the mixed dataset. By using two implementations of logistic regression, the aim was to check whether their results agreed, which was only partially the case, as it was similarly observed with two types of tree-based rules generating classifiers (CART and C5.0). To obtain a unique set of variables selected for final interpretation, the variables selected during the runs of the eight algorithms were combined in a weighted manner that took into account (i) the certainty with which each variable was part of the final set, quantified by the number of memberships in ABC set “A” during the 1000 runs described above, (ii) the performance of each classifier, as judged by its median classification accuracy or balanced accuracy in the case of unbalanced class sizes, and (iii) the uncertainty of the classification, quantified by the distance of the lower limit of the 95% CI of the classification accuracy to the level of pure guessing of 50%. The sums of these weighted memberships in ABC set “A” for each variable across all classifiers were again subjected to ABC analysis and set “A” was retained. As before [91], feature selection was repeated with the parameters not selected until classification accuracy reached the level of guessing.

To control for possible overfitting, i.e., rote learning that achieves perfect classification accuracy with a single data set but cannot classify similarly structured new data, the classification algorithms were **first** tuned with respect to the available hyperparameters. For example, the number of k in kNN was tested between 3 and 9 and the best performing variant was chosen. Similarly, the number of trees in the Random Forest was evaluated between 100 and 1600 and after it was determined that the out-of-bag error remained at a minimum of 0.02 starting at 200 trees, it was decided to use 1500 trees since, as has been shown elsewhere [92], there is no penalty for “too many” trees. SVM was used with a linear kernel since alternatives such as a radial kernel provided lower classification performance. **Second**, the analyses were performed in 1000 cross-validation runs, as described above. **Third**, a negative control condition was created by permuting the variables in the training data set. A classification that is better than chance when trained with permuted data would indicate possible overfitting. **Fourth**, several different classifiers were applied to avoid relying on a single method for the analysis, where overfitting occasionally occurred.

#### 2.3.5. Statistical Assessment of Changes in Relevant Parameters Related to Surgery Outcomes

To obtain an interpretation of the surgery-associated changes across all parameters identified as informative, the parameters selected in the previous step of data analysis were further evaluated. **First**, correlations in the changes between relevant parameters were assessed by calculating Spearman correlation coefficients ρ [93] of the differences between baseline and postoperatively recorded values and also for raw postoperative values for comparison. **Second**, patients with a postoperative reduction in CRS-related symptoms according to the relevant parameters identified in the previous analysis steps were sought from the directions of change.

The sign of change from before to after surgery was coded as (1, 0, 1) for improved, unchanged, or worsened, respectively. After standardizing the signs of improvement or deterioration across all parameters, the agreement of the direction of change between parameters was assessed using χ^2^ statistics [55]. The coded signs were summed for each subject, and from these marginal (row) sums of the sign-parameter matrix, those patients were identified for whom surgery had no consistent overall effect with respect to the relevant parameters or for whom its overall effects tended toward worsening. **Third**, the values of parameters identified as informative for changes after surgery, indicating no or mild symptoms, were used to count patients who could be considered healthy after surgery in terms of CRS symptomatology.

## 3. Results

Of the patients enrolled at baseline before surgery, one woman was excluded from data analysis because of lack of testing in most parameters and baseline and absence from the second test. Baseline data collected from the remaining *n* = 157 patients included 3611 numbers in ordinal- or interval-scale variables, of which 60 were missing, and 1256 values in nominal variables, of which 129 were missing. A total of 90 patients returned to the study at the postoperative session and provided a data set that included 1800 numbers in ordinal- or interval-scale variables, of which 32 were missing, and 180 values in nominal variables, of which 4 were missing. The reasons for the non-return of the other 67 are not known. After data preprocessing with outlier removal, imputation and one-hot transformation, the data space was complete and comprised 6840 numbers in 37 variables of which 1 was the test occasion.

**Figure 2:**
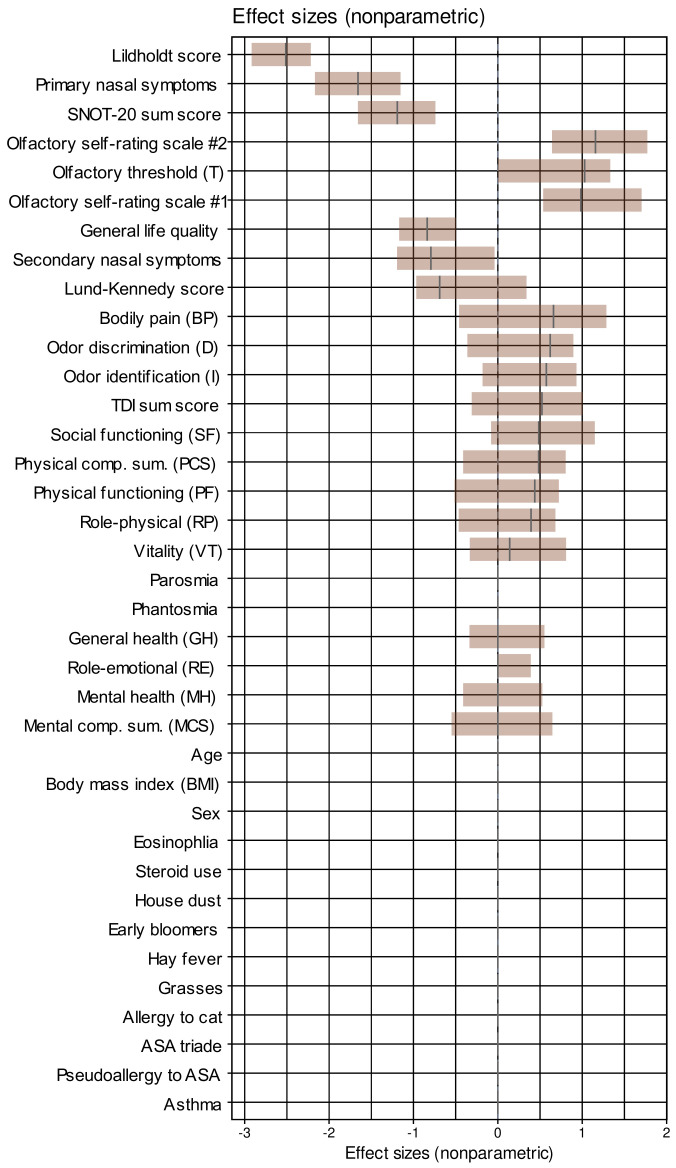
**Effect sizes of the changes from baseline to the data acquired after endoscopic nasal surgery, sorted for decreasing magnitude**. Non-parametric effect sizes were implemented as the Impact effect size measure [58]. The effect sizes are shown as black lines while the brown bars indicate the 95% confidence intervals obtained from 1000 repetitions of effect size calculation on data randomly drawn from the original data set using bootstrap resampling [60,94]. House dust, cat, grasses, and early bloomers denote allergy to the respective items. The figure has been created using the R software package (version 4.1 for Linux; https://CRAN.R-project.org/ (accessed on 16 September 2021). (R Development Core Team, 2008)) and the library “inspectdf” (https://CRAN.R-project.org/package=inspectdf (accessed on 16 September 2021) [95]).

The analyzed cohort comprised 52 men and 38 women (recovery rate 69.6%) aged 50 ± 14.92 (mean ± standard deviation) and a BMI of 27.4 ± 4.9 kg/m^2^. A summary of the main descriptive statistics of the d = 37 parameters together with the *p*-values obtained in exploratory comparisons between the two test occasions is shown in Table 1. Almost all variables differed significantly between the two test occasions. Effect sizes are shown in Figure 2.

### 3.1. Data Structures Reflecting Changes from the Pre- to the Post-Surgery State

**Figure 3:**
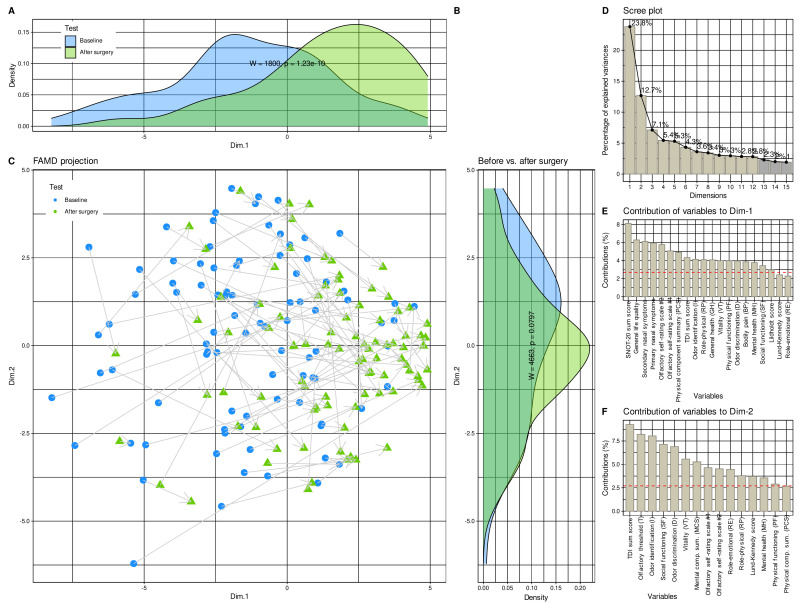
**Results of factor analysis for mixed data (FAMD) of the d = 37 variables acquired before and after the surgery**. (**A**–**C**) Data structure in the input space of d = 37 variables acquired from the patients before and four months after endoscopic nasal sinus surgery. The data structure has been obtained by means of data projection via FAMD. The plot associated with this analysis shows the sample separation in the first and second dimension of the projection (Dim.1 versus Dim.2). The marginal distribution plots show the segregation of the test occasions along the first principal and second dimensions. The *p*-values are the results from a Mann–Whitney U-test [56,57]. (**D**) Bar chart of the fraction of variance explained by the dimensions of the FAMD projection, sorted in descending order of magnitude. (**E**,**F**) Barplot of the contribution of parameters to dimensions 1 and 2 of the data projection. The dashed horizontal reference line corresponds to the expected value if the contribution where uniform. House dust, cat, grasses, and early bloomers denote allergy to the respective items. The figure has been created using the R software package (version 4.1 for Linux; https://CRAN.R-project.org/ (accessed on 16 September 2021) [47]) and the R packages “ggplot2” (https://cran.r-project.org/package=ggplot2 (accessed on 16 September 2021) [96]) and “FactoMineR” (https://cran.r-project.org/package=FactoMineR (accessed on 16 September 2021) [68]).

Unsupervised analyses of a data structure that would support a change from the pre- to the post-surgery state and implemented as factor analysis for mixed data to project the 37 × 180 (d × 2*n*) sized data space (Figure 3B), containing the information about d = 37 variables that were recorded before and after surgery or did not change between the two time points and acquired from *n* = 90 patients, yielded 12 dimensions with eigenvalues >1. The two test occasions before and after surgery were already significantly separated (Wilcoxon W = 1800, *p* = 1.29 × 10^−^^10^; Figure 3A) in the first dimension that explained 23.8% of the total variances in the data set (Figure 3D). Their separation in the second dimension, which explained 12.8% of the variance, only narrowly missed statistical significance (Figure 3C). Variables most relevantly contributing to the data projection onto the first two dimensions (Figure 3E,F) included the complete SNOT-20 score derived parameters, the olfactory parameters, as well as anatomical and pathological parameters of the nasal cavity.

### 3.2. Parameters Containing Relevant Information about Operation-Related Changes

**Figure 4:**
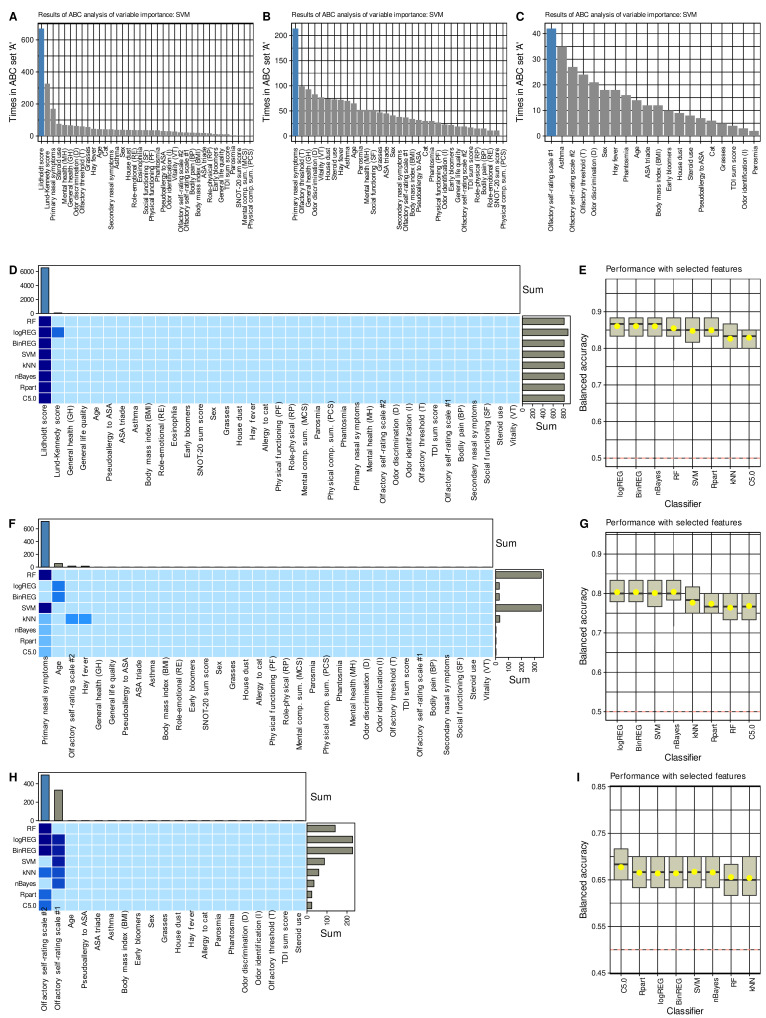
**Results of the feature selection process aimed at variables providing relevant information on changes from before to after endoscopic sinus surgery**. (**A**–**C**) Bar plots showing the number at which an item categorization technique implemented as a computed ABC analysis [72] placed the respective variables in ABC subset “A”, i.e., among the most relevant items, during the 1000 cross-validation runs on randomly selected disjoint training and test data sets, analogously to the analyses reported in [71]. The blue bars show the finally selected items based on their occurrence in the relevant subset and on the most frequent size of ABC subset “A”. The results are only shown for support vector machines (SVM) as an example for the feature selection process. (**D**,**F**,**H**) Matrix heatplot color-coding the number of runs, from the 1000 cross validations, during which the respective variable was placed in ABC subset “A”, i.e., selected as relevant for the respective algorithm to identify whether a data set instance has been acquired before or after the surgery. The feature selection was performed in three steps where in each subsequent step the parameter category from which in the previous step the relevant variables were selected was left out. More intense and darker blue colors indicate higher importance scores (for details see methods section). The bar charts above the heat matrix show the sum of placements of a variable in ABC subset “A” across all algorithms. The blue bars indicate the finally selected variables used to train the algorithm. (**E**,**G**,**I**) Boxplots of the performance of different types of machine-learning algorithms in the assignment of data set instances as acquired either before or after the surgery, using the features shown in the matrix heat plot at the left of the respective plots. The boxes have been constructed using the minimum, quartiles, median (solid line within the box), and maximum. The whiskers add 1.5 times the interquartile range (IQR) to the 75th percentile or subtract 1.5 times the IQR from the 25th percentile. The arithmetic means are additionally shown as yellow dots. House dust, cat, grasses, and early bloomers denote allergies to the respective items. The figure has been created using the R software package (version 4.1 for Linux; https://CRAN.R-project.org/ (accessed on 16 September 2021) [47]) and the R packages “ggplot2” (https://cran.r-project.org/package=ggplot2 (accessed on 16 September 2021) [96]).

After determining that the data space reflected changes induced by surgery, feature selection methods using eight different supervised algorithms and item categorization implemented as computed ABC analysis identified parameters that carried the relevant information about changes from baseline to the post-surgery examination (Figure 4). The procedure of selecting the features, i.e., the training and test parameters, quantifying the importance of each variable for the correct functioning of the algorithm, and selecting the relevant elements by ABC analysis, was carried out in three repetitions, omitting in the next repetition the category from which the most relevant features were selected in the previous step. Thus, the first analysis started with the whole set of d = 37 parameters.

The Lildholdt score was ranked first-line among the most important information by all algorithms to identify whether parameters were collected from a patient before or after surgery (Figure 4). With the Lildholdt score alone, median classification accuracies up to 86.7% (regression analyses) in the chosen cross-validation scenario were attained (Table 2). Moreover, the simple CART-derived rule “*IF* Lildholdt ≥ 1 *THEN* data has been acquired before the surgery *ELSE* data has been acquired after surgery” created once using 80% of the data provided 85% accuracy (95% CI 73.8–95%) for correctly identifying the acquisition time in a 1000 bootstrap resampling experiment performed on the remaining 20% of the data.

Omitting the endoscopic parameters and repeating the above analyses with the remaining d = 34 parameters, the second-line feature capturing changes in clinical signs or symptoms associated with surgery was the “primary nasal symptoms” derived from the SNOT-20 questionnaire. However, the regression algorithms had problems because they selected age as a relevant feature, with which they then failed to distinguish between baseline and postoperative data but succeeded when “primary nasal symptoms” was used as the majority vote across all algorithms. Thus, for primary nasal symptoms, all algorithms were again able to identify whether the parameters were collected from a patient before or after surgery with better than guesswork accuracy, i.e., accuracies including the lower limit of the 95% CIs > 50%; however, performance was not as good as for the Lildholdt score (Table 2).

Omitting the parameters related to quality of life and repeating the above analyses with the remaining d = 20 parameters, the third-line feature capturing changes in clinical signs or symptoms related to surgery was the 10-point self-rating scale #2, i.e., the scale on which subjects rated their olfactory function from 1, “not present” to 10, “excellent”. The decision between the two self-rating scales was narrow, i.e., both were placed on top by half of the algorithms. Only the performance of algorithms using rating scale #2 was decisive for the majority vote for this scale, as described in the methods section for the weighted combination of the results across algorithms. With the olfactory self-rating scale #2, all algorithms were able to detect whether the parameters had been recorded by a patient before or after surgery with better-than-guessing accuracy, i.e., accuracy > 50% including the lower limits of the 95% CIs (Table 2). However, performance approached the 50% threshold, and therefore, analyses were stopped at this point and no further relevant variables were sought among the remaining variables.

Across all classifiers, when training was performed with permuted features, none of the classifiers outperformed guessing with a classification accuracy of about 50%. This suggests that overfitting was probably not behind these observations. Moreover, the selected variables were among those that contributed to the 1st dimension of the FAMD projection of the data set, in which the test occasions were significantly separable (Figure 3C). Furthermore, the parameters chosen by the machine-learning-based feature selection were those with the largest effect sizes between the two study occasions (Figure 2), which provides a further internal validation of the results.

### 3.3. Changes in Relevant Parameters

Supervised analyses to select parameters that provide relevant information to detect changes associated with endoscopic paranasal sinus surgery identified three parameters of successive importance, starting with the first, i.e., the endoscopic Lildholdt score, and as second- and third-ranked parameters, primary nasal symptoms as a disease-specific measure of quality of life, and subjectively perceived function of one’s sense of smell. These parameters were examined to assess if they represented the same information and if they could serve as subgroup criteria for the outcome of the surgery.

**Figure 5:**
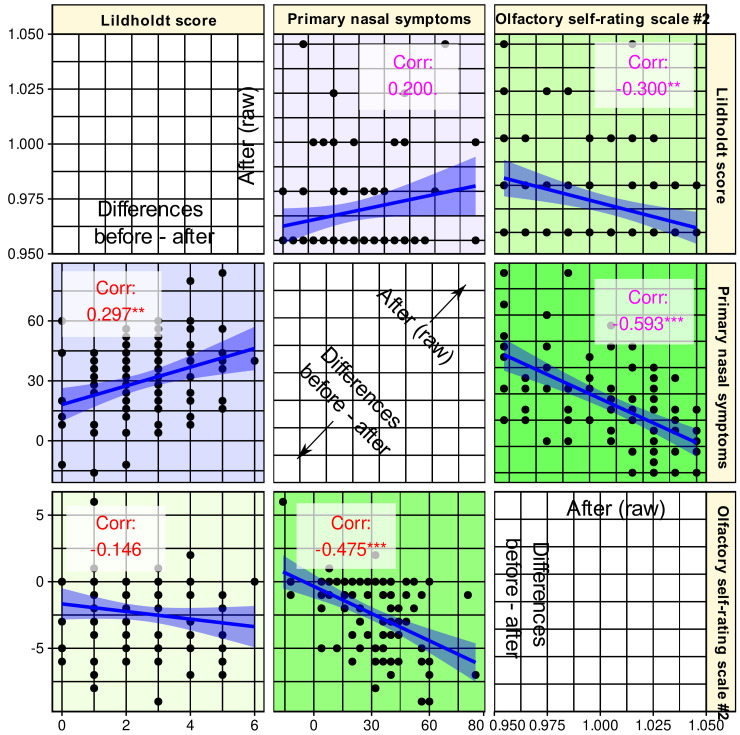
**Correlation scatterplot matrix of the parameters selected as informative for the changes accompanying endoscopic nasal sinus surgery.** The lower triangle shows the correlation of the differences between baseline and post-surgery assessments. The upper triangle shows the correlations between raw data acquired after the surgery. The regression lines (and 95% confidence intervals) are added for visual guidance; however, statistical evaluations consisted of the calculation of non-parametric Spearman’s correlation coefficients ρ, which are provided in the panels along with indicators of statistical significance: ***: *p* < 0.001, **: *p* < 0.01, *: *p* < 0.05, .: *p* < 0.1. The figure has been created using the R software package (version 4.1 for Linux; https://CRAN.R-project.org/ (16 September 2021) [47]) and the libraries “ggplot2” (https://cran.r-project.org/package=ggplot2 (accessed on 16 September 2021) [96]) and “GGally” (https://CRAN.R-project.org/package=GGally (accessed on 16 September 2021) [98]).

Changes in self-assessment of olfactory function were negatively correlated with disease-specific quality of life, which is in line with expectations as the parameters are inversely scaled. Thus, a lower value for primary nasal symptoms indicates a better quality of life, and olfactory function follows this direction (Figure 5 lower triangle). However, a value of ρ^2^ = 0.226 indicates a very weak correlation if any [97], which argues against the olfactory function simply being redundant to the parameters found in the first two searches. Furthermore, changes in olfactory function did not correlate with changes in the Lildholdt score although in the raw values acquired after the surgery, an again very weak correlation was observed (Figure 5 upper triangle).

**Figure 6:**
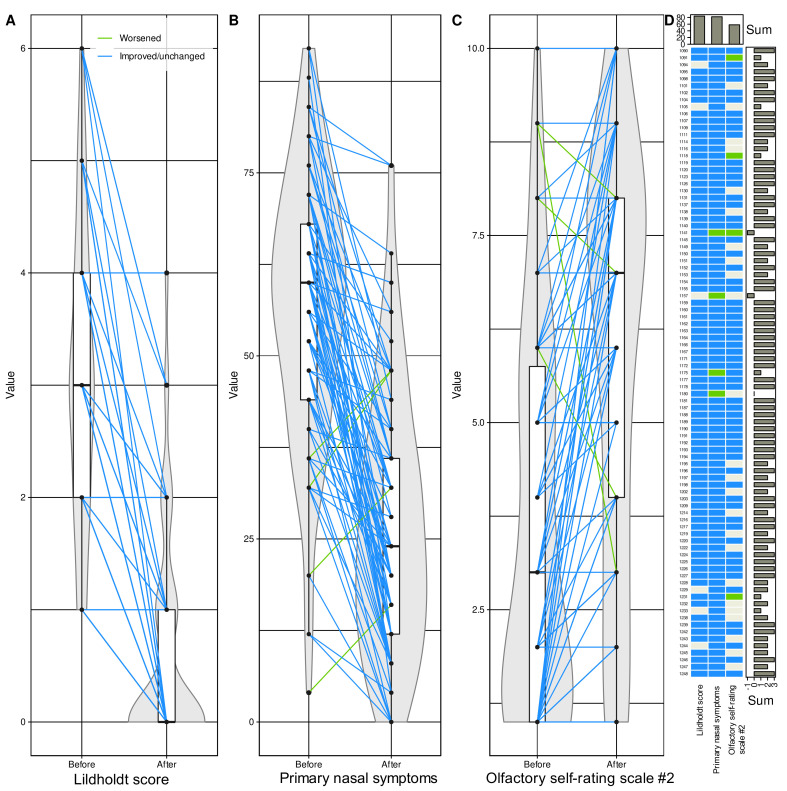
**Subset of d = 6 variables found during the machine learning-based feature selection (Figure 4) to be, out of d = 37 variables, those carrying relevant information about whether a dataset instance has been acquired before or after endoscopic nasal sinus surgery.** (**A**–**C**) Individual data points are shown as dots; please note that they may overlap for ordinally scaled variables. Individual measurements are connected by straight lines between baseline and postoperative values, colored blue for changes towards improvement of the respective parameters and colored green if signs or symptoms worsened after surgery. The points are plotted on violin plots showing the probability density distribution of the variables and are overlaid with boxplots showing more basic descriptive statistics. The boxes have been constructed using the minimum, quartiles, median (solid line within the box), and maximum. The whiskers add 1.5 times the interquartile range (IQR) to the 75th percentile or subtract 1.5 times the IQR from the 25th percentile. (**D**) Matrix heat plot color-coding the direction of change in each of the six variables in panels (**A**–**C**) from before to after surgery. The signs were adjusted to indicate improvement (blue) or no improvement (green) across all parameters. Marginal sums are shown as barplots. The figure has been created using the R software package (version 4.1 for Linux; http://CRAN.R-project.org/ (16 September 2021) [47]), and the R libraries “ggplot2” (https://cran.r-project.org/package=ggplot2 (accessed on 16 September 2021) [96]), “ggforce” (https://cran.r-project.org/package=ggforce (accessed on 16 September 2021) [99]) and “ComplexHeatmap” (https://bioconductor.org/packages/release/bioc/html/ComplexHeatmap.html (accessed on 16 September 2021) [100]).

Changes in anatomic, quality of life, or olfaction-related parameters identified as carrying relevant information about the changes associated with the surgery were mostly in the desired direction of improvement (Figure 6). This was consistently observed in the Lildholdt score, which improved in 84 patients (93.3%) while remaining unchanged in the other 6, and in quality of life (primary nasal symptoms), which improved in 86 patients (95.6%) while worsening in 4. Olfactory function was the least affected, i.e., 62 patients (68.8%) reported that their sense of smell had improved, while 24 patients had no such perception and 4 even reported a worsening. For the three main parameters characterizing changes associated with the surgery, only two patients had no overall positive effect, i.e., the parameters were unchanged or their changes for better or worse balanced each other, and changes for worse predominated in two other patients. However, because of this small number, it was not possible to perform a subgroup analysis to determine the reasons for the lack of overall improvement following surgery. An attempt to analyze baseline parameters informative of subjective olfactory function improvement using the same feature selection approach as above was completely unsuccessful.

After surgery, 59 patients had a normal Lildholdt score, corresponding to a significant increase in the number of symptom-free patients from baseline in this sign (χ^2^ = 8.7111, df (degrees of freedom) = 1, *p* = 0.003163). Regarding primary nasal symptoms, the number of symptom-free patients (*n* = 24 after surgery) did not increase significantly (χ^2^ = 0.86403, *p* = 0.3526). There is no clear cut-off value for subjectively assessed olfactory function; therefore, symptom freedom was also checked in the TDI sum score of the olfactory tests. While at baseline this indicated 43 patients with anosmia, 37 with hyposmia, and 10 patients with normal olfactory function, after the surgery these figures changed to 20, 55, and 15 patients, respectively (χ^2^ = 11.902, *p* = 0.0005606). Regardless of the category of olfactory diagnosis, the TDI sum score improved in 66 patients, whereas it decreased in 22 patients. In 15 patients, the increase was ≥ 5.5 points, which should be perceptible [28], but only in 2 of them this also coincided with normosmia after surgery.

## 4. Discussion

In this data-driven analysis, criteria were identified that were informative, in clear rank order, for changes in various clinical signs and symptoms of CRS patients associated with functional endoscopic sinus surgery. The analyses indicated that changes associated with this surgery can be summarized quite comprehensively by the Lildholdt score. A simple trained algorithm can detect with about 90% accuracy whether the score was taken before or after surgery. Even when a simple decision rule, created from a fraction of the data, is applied to data that has been completely removed from all analyses until it is only used to check predictive quality of that rule, 80% accuracy is achieved. However, the Lildholdt score is an endoscopic measure and may be too technical a piece of information for non-ENT specialists and unlikely to be appropriate or sufficient to be the only communication to a patient when discussing the success of surgery. In those cases, more information is needed, but this information should not repeat the Lildholdt score in other terms but should convey relevant further important effects of the surgery. Hence, the present complex feature selection approach that singled out parameters that meet these criteria.

Functional endoscopic nasal sinus surgery was found to influence three main categories of variables, i.e., endoscopic markers of the nasal cavity, quality of life, and sense of smell. However, among several available choices in these categories, the present analyses selected those that contained relevant information not already included in other parameters. This showed that quality of life did not change broadly and nonspecifically, as the items of the SF-36 questionnaire appeared to be uninformative, as did the general quality of life subscores of the SNOT-20 disease-specific questionnaire. In contrast, the subscore “primary nasal symptoms” of the disease-specific SNOT-20 questionnaire appeared to be more informative of changes in patients’ quality of life. However, SF-36 scores improved to a statistical extent after surgery for some items, except for the emotional and psychological subscores and the subscore indicating general health. This is roughly consistent with previous observations in CRS patients [101], up to the detail that emotional role function does not appear to be a particular facet that was improved by nasal sinus surgery, as in the referenced work a *p*-value of 0.03 would not have passed α correction (see Table 2 in [101]).

In the analyses, the sense of smell was also selected as a carrier of relevant information about the effects of surgery, especially its subjectively perceived acuity. Although olfactory function and quality of life were not completely uncorrelated, a sufficient level of nonredundancy is presumed in the only very weak correlations between the three parameters finally selected. However, the significant *p*-values of the correlations at the 0.01 or 0.001 levels (Figure 5) might suggest a discussion of some degree of interdependence of the selected parameters in addition to the endoscopic findings. Both other informative parameters reflect patients’ subjective perceptions rather than clinical test results. Of the two variants of olfactory function self-assessment, both seemed to provide fairly similar information, as the vote across the algorithms was 50/50 and only a weighting by classification success steered the decision toward scale #2. For example, SVM selected scale 1# before scale #2 (Figure 4). In the separate analyses, a combination of both scales provided better assignment of olfactory diagnostic categories [31]; however, this was not an aim in the present analyses. As separately analyzed in the *n* = 157 subjects in the present cohort in whom olfactory function had been assessed at baseline [31], self-ratings mirror olfactory function assessed with a clinical test; however, the correlation between the two is weak and, if any, stronger in women than in men. Similarly, the correlation with overall quality of life is rarely significant when the analyses are separated by olfactory diagnostic categories and patient gender. Thus, olfactory self-assessment reflects a subjective dimension of the sense of smell that cannot be attributed to a general better feeling or to measured olfactory function. This dimension seemed to be most informative for the changes associated with the present surgery. Nevertheless, the inclusion of olfaction accounts for the observation that its dysfunction is very common in CRS, with up to 78% [2,3]. In the present data set, as many as 89% of CRS patients had below-normal olfactory function at baseline, which decreased only to 83.3% after surgery but the number of subjects with anosmia was more than halved. Another indication of an importance of the sense of smell as a symptom in its own was the difference observed twice between the outcome groups of patients, although, as stated in the results section, this must be viewed with great caution because of statistical weakness. Nevertheless, better outcome seemed to be associated with better sense of smell after surgery. Given the higher information value of subjective ratings, which were preferred to changes in olfactory acuity measured by a clinical test in the present feature selection procedure, it should be kept in mind that this subjectivity may involve a bias depending on patients’ expectations of improvement after surgery. However, the associated increase in TDI score as a result of a clinical test is reassuring that olfaction was indeed an important parameter that improved after surgery. As discussed above, the preference to a particular parameter does not mean that other parameters did not change as well; they just did not provide relevant information to determine whether a record instance was acquired before or after surgery in addition to the information already provided by the selected parameters. On the other hand, clinical tests are not free from biases, such as variability due to intranasal airflow changes [102].

The present analyses aimed at information reduction, i.e., from d = 37 variables that were selected because they had previously been shown to change after sinus surgery and had indeed changed significantly in most cases, and d = 3 variables from three different categories were selected that should be sufficient to convey the relevant information about the success of this type of surgery. Thus, the analysis was “greedy,” i.e., it stopped when the included parameters provided enough information and did not exhaustively include other significant parameters because the minimal further information gain did not balance the increasing costs of more complex models. For the clinical setting, this means that if a minimalist approach is preferred, the Lildholdt score may be sufficient as an outcome marker of endoscopic nasal polyposis surgery, but if more complex information is desired, the SNOT-20 questionnaire and the perception of olfactory function should be included. The latter two emphasize the subjective component of the success of surgery, which may be important to the patient, rather than endoscopic or olfactory test results, which are meaningful to experts in the field but remain technical to others. Nevertheless, while the analysis included d = 37 variables that had been chosen to capture many clinical factettes of CRS associated with nasal polyposis associated with its surgical treatment. The predominantly data-driven approach to identifying relevant parameters that characterize the outcomes of endoscopic nasal surgery in an informative manner was largely unbiased, i.e., without a particular focus on specific variables preselected by the surgeon’s expectations. This contrasts with the ultimately too small sample size, which, although comparable to previous work [24], proved to be too low to determine parameters present at baseline before the surgery that might be meaningful to advise a patient for or against endoscopic surgery. Of note, both the unsupervised and supervised methods applied to analyze the present data set were designed to allow subgroup analyses.

The present analyses included unsupervised and supervised statistical and machine-learning methods, with the latter dominating because prior classification of data into those collected before and those collected after surgery was the main goal of the analysis. An overview of machine learning in olfactory research has been published elsewhere [103]. As mentioned in the methods section, unsupervised methods aim at pattern recognition by detecting structures in the data that could lead to clinically relevant subgroups. They have been repeatedly applied to olfaction-related data by us [104,105] and others [106].

Their use in the CRS context has been reviewed previously [107], but with a more limited focus on cluster analysis and with a restriction to hierarchical and non-hierarchical methods, of which Ward’s clustering method [108] and k-means clustering [109] are prototypes. More modern methods of cluster detection include emergent self-organizing maps of artificial neurons [110], hierarchical density-based spatial clustering of applications with noise (DBSCAN) [111], adaptive density peak clustering [112], and others. In the present dataset, detection of new structures was not the main focus, but an exploratory analysis using a combination of the above classical clustering methods according to the workflow proposed in [69] indicated a relevance of subjectively perceived olfactory function for subgrouping; however, since this did not lead further than the presently reported results, it was abandoned. In contrast, the present analyses were dominated by supervised algorithms, i.e., classification algorithms that are trained with a subset of the data, including class information, i.e., information about when a record instance was acquired, to learn the class label assignment from the provided information. The class assignment performance is monitored during learning, since it is known in the training data set and the algorithms can thus be corrected during training. The subsequent task for a trained algorithm is then to apply the learned rules for assigning a class label from given information to new data that does not have a class label. The latter was only subsequently used to measure the classification performance reported here. As unsupervised methods, supervised methods of machine learning have been repeatedly applied to olfaction-related data by us [104,113] and others [114].

## 5. Conclusions

The present analyses provided a ranked proposal of criteria against which to evaluate the consequences of functional endoscopic sinus surgery in CRS patients with nasal polyposis. The criteria capture three different facets of this clinical setting. The most informative criterion was the Lildholdt score as an endoscopic measure. If the success of surgery is measured by this criterion alone, complete remission of symptoms can be expected in 65.6% of patients. However, if it is considered that it is not sufficient to tell the patient that the relevant endoscopic score is now normal, the overall benefit of endoscopic surgery for nasal polyposis has additional components that have a subjectively perceived impact on patients’ daily lives. That is, further relevant information comes from responses to a disease-specific quality of life questionnaire (SNOT-20) and patients’ perceptions of their own sense of smell. Patients cannot expect a complete return to normal on all relevant parameters, but may be left with residual symptoms such as impaired quality of life or lower than normal olfactory function as measured by a clinical test. Overall, endoscopic sinus surgery resulted in improvement of relevant clinical signs and symptoms in 95% of patients, while worsening was very rare and occurred in 2% of patients.

## Figures and Tables

**Table 1:** **Basic descriptive statistics of d = 37 parameters of four categories related to (i) nasal anatomy, (ii) olfaction, (iii) quality of life, and (iv) demography or concomitant diseases and allergies affecting nasal function**. If acquired before or after the surgery, differences were explored using Wilcoxon–Mann–Whitney U tests for interval or ordinal variables and χ^2^ tests for binary (yes/no) or categorial variables. Only *p*-values are shown for these explorative analyses.

Parameter Category	Test Battery	Parameter	Mean ± SD/*n* Baseline	Range Baseline	Mean ± SD/*n* Post-Surgery	Range Post-Surgery	Wilcoxon/χ^2^ *p*-Value
**Nasal anatomy/pathology**		Lildholdt score	3.08 ± 1.32	1–6	0.58 ± 0.96	0–4	9.115 × 10^−25^
		Lund–Kennedy score	7.41 ± 3.22	1–18	5.76 ± 4.16	0–18	0.0002092
		Eosinophilia in nasal tissue	1.39 ± 1.11	0–3	same		-
**Olfactory**	Sniffn Sticks	TDI sum score	17.84 ± 9.66	2–35.5	22.8 ± 8.24	5–41.75	0.0006678
		Olfactory threshold (T)	0.59 ± 0.74	0–2.3	0.97 ± 0.77	0–2.69	0.0003911
		Odor discrimination (D)	7.83 ± 4.15	0–16	9.48 ± 3.42	1–16	0.004951
		Odor identification (I)	7.54 ± 4.36	0–15	9.8 ± 3.68	1–15	0.0004697
		Olfactory self-rating scale #1	1.88 ± 1.64	0–7	3.48 ± 1.72	0–7	4.367 × 10^−9^
		Olfactory self-rating scale #2	3.53 ± 2.51	1–10	5.91 ± 2.77	1–10	3.669 × 10^−8^
		Parosmia	6		4		0.7449
		Phantosmia	7		4		0.5337
**Quality of life**	SNOT-20	Primary nasal symptoms	56.44 ± 18.15	4–92	26.62 ± 17.55	0–76	3.331 × 10^−18^
		Secondary nasal symptoms	31.44 ± 18.69	0–80	18.26 ± 15.04	0–63.33	9.597 × 10^−7^
		General life quality	33.98 ± 19.94	0–88.89	18.81 ± 15.98	0–62.22	1.838 × 10^−7^
		SNOT-20 sum score	38.83 ± 16.05	5–84	20.6 ± 13.66	1–62	1.516 × 10^−12^
	SF-36	Physical functioning (PF)	77.61 ± 22.49	10–100	86.56 ± 19.13	10–100	0.0005252
		Role-physical (RP)	70 ± 38.62	0–100	84.44 ± 31.93	0–100	0.004417
		Bodily pain (BP)	72.83 ± 24.68	10–100	82.56 ± 24.8	0–100	0.002408
		General health (GH)	56.22 ± 19.86	5–100	61.11 ± 20.67	15–100	0.1172
		Vitality (VT)	51.83 ± 19.39	10–100	60.56 ± 17.13	25–95	0.001698
		Social functioning (SF)	79.31 ± 20.28	12.5–100	88.47 ± 15.81	37.5–100	0.001161
		Role-emotional (RE)	78.89 ± 37.54	0–100	87.41 ± 29.38	0–100	0.1271
		Mental health (MH)	72.76 ± 16.26	36–100	76 ± 15.39	32–100	0.201
		Physical component summary (PCS)	64.34 ± 23.99	8.4–102.58	75.34 ± 23.42	4.56–108.72	0.0004798
		Mental component summary (MCS)	68.69 ± 21.89	0.95–97.86	73.62 ± 19.91	−1.53–104.69	0.1335
**Demographic/concomitant disease-related**		Age	50.5 ± 14.92	13.9–82.5	50.83 ± 14.92	14.6–82.83	-
		Body mass index (BMI)	27.37 ± 4.88	20.42–44.08	same		-
		Sex	52/38		same		-
		Steroid use	71		same		-
		Allergy to house dust	8		same		-
		Allergy to early bloomers	1		same		-
		Allergy to hay fever	17		same		-
		Allergy to grasses	3		same		-
		Allergy to cat	2		same		-
		Asthma, pseudoallergy to acetylsalicylic acid and nasal polyps	6		same		-
		Pseudoallergy to acetylsalicylic acid	9		same		-
		Asthma	26		same		-

**Table 2:** **Classification performance measures for correctly identifying whether a record was acquired before or after endoscopic sinus surgery are obtained when training different classifiers, i.e., random forests (RF), logistic regression in two implementations (logReg, binReg), support vector machines (SVM), k-nearest neighbors (kNN), naïve Bayes, classification and regression trees (CART), and a hierarchical rule based C5.0 classifier with the information from d = 37 variables used in Table 1.** The results represent the medians and 95% confidence intervals of the performance measures obtained during 1000 runs using a class-proportional random sampling of the data set into disjoint training (2/3 of the data set) and test data subsets (1/3). The classifiers were trained with the full information in d = 37 variables and with the features identified as informative for the task to identify whether a data set instance had been acquired before or after the surgery (Lildholdt score only, primary nasal symptoms only, or olfactory self-rating scale #2 values only; see Figure 4). Note that class sizes were equal; therefore, accuracy and not balanced accuracy is adequate.

Classifier	RF				logReg			
**Feature set**	Full	Lildholdt only	Primary nasal symptoms only	Olfactory self-rating scale #2 only	Full	Lildholdt only	Primary nasal symptoms only	Olfactory self-rating scale #2 only
**Sensitivity, recall**	83.3 (69.9–96.7)	86.7 (80–100)	76.7 (60–90)	76.7 (50–93.3)	83.3 (66.7–96.7)	86.7 (80–96.7)	80 (66.7–93.3)	63.3 (50–80.1)
**Specificity**	86.7 (73.3–96.7)	83.3 (60–93.3)	76.7 (53.3–93.3)	53.3 (36.7–73.3)	83.3 (66.7–96.7)	83.3 (73.3–93.3)	80 (66.7–90)	70 (53.3–80)
**Positive predictive value, precision**	85.7 (74.3–96)	83.9 (71.4–93.3)	76.9 (64.5–91.3)	62.8 (54.8–71.9)	83.9 (73–95.8)	84.4 (77.1–93.3)	80.8 (70.3–90)	66.7 (56.5–77.8)
**Negative predictive value**	83.9 (73.3–96)	87.1 (79.3–100)	76.7 (66.7–88.5)	70.6 (55.6–88.9)	83.9 (71.9–96.2)	87.1 (79.3–96.3)	80 (70.7–92.3)	65.6 (56.3–77.4)
**F1**	84.2 (74.6–91.8)	85.7 (78.7–93.1)	76.7 (66.7–85.2)	69.4 (55.2–78.9)	83.9 (73.1–91.8)	86.2 (79.3–93.3)	80 (71.2–88.5)	65.5 (54.5–76.7)
**Accuracy**	85 (75–91.7)	85 (78.3–93.3)	76.7 (66.7–85)	65 (55–75)	83.3 (73.3–91.7)	86.7 (78.3–93.3)	80 (71.7–88.3)	66.7 (56.7–76.7)
**ROC-AUC**	92.1 (85.2–97.5)	92.8 (87–97.6)	84.3 (75.4–92.7)	69.8 (58.6–80.2)	86.4 (76.8–92.9)	93.4 (88.2–97.5)	87.7 (79.4–94.6)	73.3 (62.8–83.7)
**Classifier**	**binReg**				**SVM**			
**Feature set**	Full	Lildholdt only	Primary nasal symptoms only	Olfactory self-rating scale #2 only	Full	Lildholdt only	Primary nasal symptoms only	Olfactory self-rating scale #2 only
**Sensitivity, recall**	83.3 (63.3–93.4)	86.7 (80–96.7)	80 (66.7–93.3)	63.3 (50–80.1)	86.7 (66.7–96.7)	86.7 (56.7–100)	80 (66.7–93.3)	66.7 (50–83.3)
**Specificity**	83.3 (66.7–96.7)	83.3 (73.3–93.3)	80 (66.7–90)	70 (53.3–80)	86.7 (70–96.7)	83.3 (56.7–96.7)	80 (66.7–90)	66.7 (50–80)
**Positive predictive value, precision**	83.3 (71.4–95)	84.4 (77.1–93.3)	80.8 (70.3–90)	66.7 (56.5–77.8)	84.8 (74.3–95.8)	84.8 (69.8–96.3)	80.6 (69.7–89.7)	66.7 (56.8–77.8)
**Negative predictive value**	82.1 (69.7–93.6)	87.1 (79.3–96.3)	80 (70.7–92.3)	65.6 (56.3–77.4)	85.2 (73.5–96.3)	87.1 (68.4–100)	80.6 (70.3–92.3)	66.7 (57.1–79.2)
**F1**	82.6 (70.6–90.3)	86.2 (79.3–93.3)	80 (71.2–88.5)	65.5 (54.5–76.7)	84.8 (75–92.3)	85.7 (69.4–93.3)	80 (70.6–88.9)	66.7 (54.9–76.9)
**Accuracy**	83.3 (71.7–90)	86.7 (78.3–93.3)	80 (71.7–88.3)	66.7 (56.7–76.7)	85 (75–91.7)	85 (75–93.3)	80 (71.7–88.3)	66.7 (58.3–76.7)
**ROC-AUC**	86.4 (76.7–93.8)	93.4 (88.2–97.5)	87.7 (79.4–94.6)	73.3 (62.8–83.7)	85 (75–91.7)	85 (75–93.3)	80 (71.7–88.3)	66.7 (58.3–76.7)
**Classifier**	**kNN**				**Naïve Bayes**			
**Feature set**	Full	Lildholdt only	Primary nasal symptoms only	Olfactory self-rating scale #2 only	Full	Lildholdt only	Primary nasal symptoms only	Olfactory self-rating scale #2 only
**Sensitivity, recall**	76.7 (60–90)	83.3 (56.7–100)	76.7 (63.3–93.3)	70 (43.3–93.3)	76.7 (43.3–96.7)	86.7 (80–96.7)	80 (66.7–93.3)	66.7 (50–86.7)
**Specificity**	73.3 (56.7–86.7)	83.3 (56.7–100)	80 (56.7–93.3)	60 (40–80)	73.3 (43.3–93.3)	83.3 (73.3–93.3)	80 (66.7–90)	66.7 (50–80)
**Positive predictive value, precision**	73.5 (62.9–84.4)	83.9 (68.2–100)	78.6 (65.8–92)	64 (53.3–75.9)	74.3 (60.9–90)	84.4 (77.1–93.3)	81.3 (70.6–89.7)	66.7 (56.5–76.9)
**Negative predictive value**	74.6 (63.3–88)	84.4 (68.8–100)	77.4 (67.7–90.3)	67.9 (53.1–86.4)	75.9 (59.5–95)	87.1 (79.3–96.3)	80 (71–92)	66.7 (56.7–81.8)
**F1**	74.2 (63–83.9)	83.3 (69.4–91.8)	77.5 (67.9–86.2)	67.7 (49.1–78.3)	75 (54.9–85.2)	86.2 (78.7–93.3)	80 (71.4–88.9)	66.7 (54.5–77.6)
**Accuracy**	73.3 (63.3–83.3)	83.3 (71.7–91.7)	78.3 (68.3–86.7)	65 (53.3–75)	75 (61.7–85)	86.7 (78.3–93.3)	80 (71.7–88.3)	66.7 (56.7–76.7)
**ROC-AUC**	80.1 (70.3–89.4)	90.3 (79.7–96.3)	84.5 (75.6–92.5)	67.5 (55.4–78.9)	82.6 (70.4–91.3)	93.4 (88.2–97.5)	87.7 (79.4–94.6)	73.3 (62.8–83.7)
**Classifier**	**CART**				**C5.0**			
**Feature set**	Full	Lildholdt only	Primary nasal symptoms only	Olfactory self-rating scale #2 only	Full	Lildholdt only	Primary nasal symptoms only	Olfactory self-rating scale #2 only
**Sensitivity, recall**	83.3 (66.7–96.7)	86.7 (76.7–100)	76.7 (59.9–93.3)	83.3 (46.7–93.3)	85 (70–96.7)	90 (63.3–100)	76.7 (56.7–96.7)	86.7 (49.9–96.7)
**Specificity**	83.3 (70–93.3)	83.3 (56.7–93.3)	80 (53.3–96.7)	53.3 (36.7–73.3)	83.3 (66.7–96.7)	80 (53.3–93.3)	76.7 (50–96.7)	53.3 (36.7–73.3)
**Positive predictive value, precision**	82.8 (72.4–93.3)	83.9 (69.8–93.1)	79.3 (65.8–95)	63 (55.5–72)	83.3 (72.2–95.7)	80.6 (68.1–92.3)	78.1 (64.1–94.7)	63.4 (55.8–72.2)
**Negative predictive value**	83.9 (71.4–95.8)	87.1 (78.8–100)	76.7 (67.5–90.9)	75 (55.6–90.5)	84.6 (73–96)	89.3 (71.8–100)	77.1 (67.5–93.8)	77.8 (56.4–92.9)
**F1**	83.3 (72.7–90.3)	85.2 (78.7–92.1)	76.9 (67.8–86.2)	71.2 (52.6–80)	83.6 (74.2–91.2)	84.5 (73.5–90)	76.9 (67.9–84.7)	72.9 (54.5–80)
**Accuracy**	83.3 (73.3–90)	85 (76.7–91.7)	76.7 (68.3–86.7)	66.7 (55–76.7)	83.3 (75–91.7)	83.3 (75–90)	76.7 (68.3–85)	68.3 (56.7–76.7)
**ROC-AUC**	85 (73.2–93.4)	89 (76.7–96.9)	80 (70–91.6)	68.3 (58.3–77.8)	91.6 (83.2–96.8)	83.3 (75–95.2)	76.7 (68.3–85)	68.3 (58.3–76.7)

## Data Availability

Data available on request from the last author.

## References

[1] Orlandi R.R., Kingdom T.T., Smith T.L., Bleier B., DeConde A., Luong A.U., Poetker D.M., Soler Z., Welch K.C., Wise S.K. (2021). International consensus statement on allergy and rhinology: Rhinosinusitis 2021. Int. Forum Allergy Rhinol..

[2] Kohli P., Naik A.N., Harruff E.E., Nguyen S.A., Schlosser R.J., Soler Z.M. (2017). The prevalence of olfactory dysfunction in chronic rhinosinusitis. Laryngoscope.

[3] Litvack J.R., Mace J.C., Smith T.L. (2009). Olfactory function and disease severity in chronic rhinosinusitis. Am. J. Rhinol. Allergy.

[4] Pfaar O., Landis B.N., Frasnelli J., Hüttenbrink K.B., Hummel T. (2006). Mechanical obstruction of the olfactory cleft reveals differences between orthonasal and retronasal olfactory functions. Chem. Senses.

[5] Lane A.P., Turner J., May L., Reed R. (2010). A genetic model of chronic rhinosinusitis-associated olfactory inflammation reveals reversible functional impairment and dramatic neuroepithelial reorganization. J. Neurosci..

[6] Yee K.K., Pribitkin E.A., Cowart B.J., Vainius A.A., Klock C.T., Rosen D., Feng P., McLean J., Hahn C.G., Rawson N.E. (2010). Neuropathology of the olfactory mucosa in chronic rhinosinusitis. Am. J. Rhinol. Allergy.

[7] Banglawala S.M., Oyer S.L., Lohia S., Psaltis A.J., Soler Z.M., Schlosser R.J. (2014). Olfactory outcomes in chronic rhinosinusitis with nasal polyposis after medical treatments: A systematic review and meta-analysis. Int. Forum Allergy Rhinol..

[8] Le P.T., Soler Z.M., Jones R., Mattos J.L., Nguyen S.A., Schlosser R.J. (2018). Systematic Review and Meta-analysis of SNOT-22 Outcomes after Surgery for Chronic Rhinosinusitis with Nasal Polyposis. Otolaryngol. Head Neck Surg..

[9] Andrews P.J., Poirrier A.L., Lund V.J., Choi D. (2016). Outcomes in endoscopic sinus surgery: Olfaction, nose scale and quality of life in a prospective cohort study. Clin. Otolaryngol..

[10] Lind H., Joergensen G., Lange B., Svendstrup F., Kjeldsen A.D. (2016). Efficacy of ESS in chronic rhinosinusitis with and without nasal polyposis: A Danish cohort study. Eur. Arch. Otorhinolaryngol..

[11] Zhao R., Chen K., Tang Y. (2021). Olfactory changes after endoscopic sinus surgery for chronic rhinosinusitis: A meta-analysis. Clin. Otolaryngol..

[12] Zou L.Q., Hummel T., Otte M.S., Bitter T., Besser G., Mueller C.A., Welge-Lussen A., Bulut O.C., Goktas O., Negoias S. (2021). Association between olfactory function and quality of life in patients with olfactory disorders: A multicenter study in over 760 participants. Rhinology.

[13] Elkholi S.M.A., Abdelwahab M.K., Abdelhafeez M. (2021). Impact of the smell loss on the quality of life and adopted coping strategies in COVID-19 patients. Eur. Arch. Otorhinolaryngol..

[14] Smeets M.A.M., Veldhuizen M.G., Galle S., Gouweloos J., de Haan A.J.A., Vernooij J., Visscher F., Kroeze J.H.A. (2009). Sense of smell disorder and health-related quality of life. Rehabil. Psychol..

[15] Katotomichelakis M., Simopoulos E., Tripsianis G., Balatsouras D., Danielides G., Kourousis C., Livaditis M., Danielides V. (2014). Predictors of quality of life outcomes in chronic rhinosinusitis after sinus surgery. Eur. Arch. Otorhinolaryngol..

[16] Lötsch J. (2021). Biomedinformatics: A New Journal for the New Decade to Publish Biomedical Informatics Research. BioMedInformatics.

[17] Guyon I., Elisseeff A. (2003). An introduction to variable and feature selection. J. Mach. Learn. Res..

[18] Fokkens W.J., Lund V.J., Hopkins C., Hellings P.W., Kern R., Reitsma S., Toppila-Salmi S., Bernal-Sprekelsen M., Mullol J., Alobid I. (2020). European Position Paper on Rhinosinusitis and Nasal Polyps 2020. Rhinology.

[19] Nguyen D.T., Nguyen-Thi P.L., Jankowski R. (2012). How does measured olfactory function correlate with self-ratings of the sense of smell in patients with nasal polyposis?. Laryngoscope.

[20] Lund V.J., Kennedy D.W. (1995). Quantification for staging sinusitis. The Staging and Therapy Group. Ann. Otol. Rhinol. Laryngol. Suppl..

[21] Nathan K., Majhi S.K., Bhardwaj R., Gupta A., Ponnusamy S., Basu C., Kaushal A. (2021). The Role of Diagnostic Nasal Endoscopy and a Computed Tomography Scan (Nose and PNS) in the Assessment of Chronic Rhinosinusitis: A Comparative Evaluation of the Two Techniques. Sinusitis.

[22] Lildholdt T., Rundcrantz H., Lindqvist N. (1995). Efficacy of topical corticosteroid powder for nasal polyps: A double-blind, placebo-controlled study of budesonide. Clin. Otolaryngol..

[23] Bachert C., Holtappels G. (2015). Pathophysiology of chronic rhinosinusitis, pharmaceutical therapy options. GMS Curr. Top. Otorhinolaryngol. Head Neck Surg..

[24] Mattos J.L., Soler Z.M., Schlosser R.J., Mace J.C., Alt J.A., Ramakrishnan V.R., Payne S.C., Smith T.L., Beswick D.M. (2021). Olfactory Function After Surgical Treatment of CRS: A Comparison of CRS Patients to Healthy Controls. Am. J. Rhinol. Allergy.

[25] Kobal G., Hummel T., Sekinger B., Barz S., Roscher S., Wolf S.R. (1996). “Sniffin’ Sticks”: Screening of olfactory performance. Rhinology.

[26] Hummel T., Sekinger B., Wolf S.R., Pauli E., Kobal G. (1997). ‘Sniffin’ sticks’: Olfactory performance assessed by the combined testing of odor identification, odor discrimination and olfactory threshold. Chem. Senses.

[27] Oleszkiewicz A., Schriever V.A., Croy I., Hahner A., Hummel T. (2019). Updated Sniffin’ Sticks normative data based on an extended sample of 9139 subjects. Eur. Arch. Otorhinolaryngol..

[28] Gudziol V., Lötsch J., Hahner A., Zahnert T., Hummel T. (2006). Clinical significance of results from olfactory testing. Laryngoscope.

[29] Lötsch J., Hummel T. (2019). Clinical usefulness of self-rated olfactory performance—A data science-based assessment of 6000 patients. Chem. Senses.

[30] Bogdanov V., Walliczek-Dworschak U., Whitcroft K.L., Landis B.N., Hummel T. (2020). Response to Glucocorticosteroids Predicts Olfactory Outcome After ESS in Chronic Rhinosinusitis. Laryngoscope.

[31] Lötsch J., Hintschich C.A., Petridis P., Pade J., Hummel T. (2021). Self-Ratings of Olfactory Function and Their Relation to Olfactory Test Scores. A Data Science-Based Analysis in Patients with Nasal Polyposis. Appl. Sci..

[32] Jefferson M. (1961). Anosmia and parosmia. Practitioner.

[33] Fikentscher R., Rasinski C. (1986). [Parosmias--definition and clinical picture]. Laryngol. Rhinol. Otol..

[34] Landis B.N., Frasnelli J., Croy I., Hummel T. (2010). Evaluating the clinical usefulness of structured questions in parosmia assessment. Laryngoscope.

[35] Piccirillo J.F., Merritt M.G., Richards M.L. (2002). Psychometric and clinimetric validity of the 20-Item Sino-Nasal Outcome Test (SNOT-20). Otolaryngol. Head Neck Surg..

[36] Baumann I., Plinkert P.K., De Maddalena H. (2008). Development of a grading scale for the Sino-Nasal Outcome Test-20 German Adapted Version (SNOT-20 GAV). Hno.

[37] Ellert U., Bellach B.M. (1999). The SF-36 in the Federal Health Survey--description of a current normal sample. Gesundheitswesen.

[38] Ware J.E., Sherbourne C.D. (1992). The MOS 36-item short-form health survey (SF-36). I. Conceptual framework and item selection. Med. Care.

[39] Ware J.E., Spilker B. (1996). The SF-36 Health Survey. Quality of Life and Pharmaeconomics in Clinical Trials.

[40] Ware J.E. (2000). SF-36 health survey update. Spine.

[41] Brazier J.E., Harper R., Jones N.M., O’Cathain A., Thomas K.J., Usherwood T., Westlake L. (1992). Validating the SF-36 health survey questionnaire: New outcome measure for primary care. BMJ.

[42] Hays R.D., Shapiro M.F. (1992). An overview of generic health-related quality of life measures for HIV research. Qual. Life Res..

[43] Ware J.E., Kosinski M. SF-36 Physical & Mental Health Summary Scales: A Manual for Users of Version 1.

[44] Wilson D., Parsons J., Tucker G. (2000). The SF-36 summary scales: Problems and solutions. Sozial und Präventivmedizin.

[45] Lötsch J., Ultsch A. (2020). Random Forests Followed by Computed ABC Analysis as a Feature Selection Method for Machine Learning in Biomedical Data. Advanced Studies in Classification and Data Science.

[46] Ihaka R., Gentleman R. (1996). R: A Language for Data Analysis and Graphics. J. Comput. Graph. Stat..

[47] R Development Core Team (2008). R: A Language and Environment for Statistical Computing.

[48] Tukey J.W. (1977). Exploratory Data Analysis.

[49] Smirnov N. (1948). Table for Estimating the Goodness of Fit of Empirical Distributions. Ann. Math. Stat..

[50] Grubbs F.E. (1950). Sample Criteria for Testing Outlying Observations. Ann. Math. Stat..

[51] Komsta L. (2011). Outliers: Tests for Outliers. https://CRAN.R-project.org/package=outliers.

[52] Ho T.K. Random decision forests. Proceedings of the Third International Conference on Document Analysis and Recognition.

[53] Breiman L. (2001). Random Forests. Mach. Learn..

[54] van Buuren S., Groothuis-Oudshoorn K. (2011). mice: Multivariate Imputation by Chained Equations in R. J. Stat. Softw..

[55] Pearson K. (1900). On the criterion that a given system of deviations from the probable in the case of a correlated system of variables is such that it can be reasonably supposed to have arisen from random sampling. Philos. Mag. Ser. 5.

[56] Mann H.B., Whitney D.R. (1947). On a test of whether one of two random variables is stochastically larger than the other. Ann. Math. Stat..

[57] Wilcoxon F. (1945). Individual comparisons by ranking methods. Biometrics.

[58] Lötsch J., Ultsch A. (2020). A non-parametric effect-size measure capturing changes in central tendency and data distribution shape. PLoS ONE.

[59] Cohen J. (1988). Statistical Power Analysis for the Behavioral Sciences.

[60] Efron B., Tibshirani R.J. (1995). An introduction to the Bootstrap.

[61] Pagès J. (2004). Analyse factorielle de données mixtes. Revue de Statistique Appliquée.

[62] Hotelling H. (1933). Analysis of a complex of statistical variables into principal components. J. Educ. Psychol..

[63] Pearson K. (1901). LIII. On lines and planes of closest fit to systems of points in space. Lond. Edinb. Dublin Philos. Mag. J. Sci..

[64] Hirschfeld H.O. (1935). A connection between correlation and contingency. Proc. Math. Camb. Philos. Soc..

[65] Horst P. (1935). Measuring complex attitudes. J. Social. Psychol..

[66] Kaiser H.F. (1958). The varimax criterion for analytic rotation in factor analysis. Psychometrika.

[67] Guttman L. (1954). Some necessary conditions for common factor analysis. Psychometrika.

[68] Le S., Josse J., Husson F.C. (2008). FactoMineR: A Package for Multivariate Analysis. J. Stat. Softw..

[69] Kassambara A. (2017). Practical Guide To Principal Component Methods in R: PCA, M(CA), FAMD, MFA, HCPC, Factoextra; CreateSpace Independent Publishing Platform, Sthda. https://CRAN.R-project.org/package=factoextra.

[70] Kassambara A., Mundt F. (2020). Factoextra: Extract and Visualize the Results of Multivariate Data Analyses. https://cran.r-project.org/web/packages/factoextra/index.html.

[71] Lötsch J., Malkusch S. (2021). Interpretation of cluster structures in pain-related phenotype data using explainable artificial intelligence (XAI). Eur. J. Pain.

[72] Ultsch A., Lötsch J. (2015). Computed ABC Analysis for Rational Selection of Most Informative Variables in Multivariate Data. PLoS ONE.

[73] Juran J.M. (1975). The non-Pareto principle; Mea culpa. Qual. Prog..

[74] Lee J.S., Paintsil E., Gopalakrishnan V., Ghebremichael M. (2019). A comparison of machine learning techniques for classification of HIV patients with antiretroviral therapy-induced mitochondrial toxicity from those without mitochondrial toxicity. BMC Med. Res. Methodol..

[75] Good P.I. (2006). Resampling Methods: A Practical Guide to Data Analysis.

[76] Tillé Y., Matei A. (2016). Sampling: Survey Sampling. https://CRAN.R-project.org/package=sampling.

[77] Altman D.G., Bland J.M. (1994). Diagnostic tests. 1: Sensitivity and specificity. BMJ.

[78] Altman D.G., Bland J.M. (1994). Diagnostic tests 2: Predictive values. BMJ.

[79] Sørensen T.J. (1948). A Method of Establishing Groups of Equal Amplitude in Plant Sociology Based on Similarity of Species Content and Its Application to Analyses of the Vegetation on Danish Commons.

[80] Jardine N., van Rijsbergen C.J. (1971). The use of hierarchic clustering in information retrieval. Inf. Storage Retr..

[81] Brodersen K.H., Ong C.S., Stephan K.E., Buhmann J.M. The Balanced Accuracy and Its Posterior Distribution. Proceedings of the Pattern Recognition (ICPR), 2010 20th International Conference.

[82] Kuhn M. (2015). Caret: Classification and Regression Training. Astrophys. Source Code Libr..

[83] Robin X., Turck N., Hainard A., Tiberti N., Lisacek F., Sanchez J.-C., Müller M. (2011). pROC: An open-source package for R and S+ to analyze and compare ROC curves. BMC Bioinform..

[84] Liaw A., Wiener M. (2002). Classification and Regression by randomForest. R News.

[85] Venables W.N., Ripley B.D. (2002). Modern Applied Statistics with S.

[86] Meyer D., Dimitriadou E., Hornik K., Weingessel A., Leisch F. (2019). e1071: Misc Functions of the Department of Statistics, Probability Theory Group (Formerly: E1071), TU Wien. https://CRAN.R-project.org/package=e1071.

[87] Cortes C., Vapnik V. (1995). Support-Vector Networks. Mach. Learn..

[88] Cover T., Hart P. (1967). Nearest neighbor pattern classification. IEEE Trans. Inf. Theor..

[89] Mouselimis L. (2021). KernelKnn: Kernel k Nearest Neighbors. https://cran.r-project.org/web/packages/KernelKnn/KernelKnn.pdf.

[90] Bayes M., Price M. (1763). An Essay towards Solving a Problem in the Doctrine of Chances. By the Late Rev. Mr. Bayes, F.R.S. Communicated by Mr. Price, in a Letter to John Canton, A.M.F.R.S. Philos. Trans..

[91] Lotsch J., Alfredsson L., Lampa J. (2019). Machine-learning based knowledge discovery in rheumatoid arthritis related registry data to identify predictors of persistent pain. Pain.

[92] Svetnik V., Liaw A., Tong C., Culberson J.C., Sheridan R.P., Feuston B.P. (2003). Random Forest:  A Classification and Regression Tool for Compound Classification and QSAR Modeling. J. Chem. Inf. Comput. Sci..

[93] Spearman C. (1904). The proof and measurement of association between two things. Am. J. Psychol..

[94] Thomas J.D., Bradley E. (1996). Bootstrap confidence intervals. Stat. Sci..

[95] Rushworth A. (2021). Inspectdf: Inspection, Comparison and Visualisation of Data Frames.

[96] Wickham H. (2009). ggplot2: Elegant Graphics for Data Analysis.

[97] Moore D.S., Notz W., Fligner M.A. (2013). The Basic Practice of Statistics.

[98] Schloerke B., Crowley J., Cook D., Briatte F., Marbach M., Thoen E., Elberg A., Larmarange J. (2018). GGally: Extension to ‘ggplot2’.

[99] Pedersen T.L. (2020). ggforce: Accelerating ‘ggplot2’. https://CRAN.R-project.org/package=ggforce.

[100] Gu Z., Eils R., Schlesner M. (2016). Complex heatmaps reveal patterns and correlations in multidimensional genomic data. Bioinformatics.

[101] Baumann I., Blumenstock G., Praetorius M., Sittel C., Piccirillo J.F., Plinkert P.K. (2006). [Patients with chronic rhinosinusitis: Disease-specific and general health-related quality of life]. Hno.

[102] Landis B.N., Hummel T., Hugentobler M., Giger R., Lacroix J.S. (2003). Ratings of overall olfactory function. Chem. Senses.

[103] Lotsch J., Kringel D., Hummel T. (2019). Machine learning in human olfactory research. Chem. Senses.

[104] Lötsch J., Hummel T., Ultsch A. (2016). Machine-learned pattern identification in olfactory subtest results. Sci. Rep..

[105] Lötsch J., Reither N., Bogdanov V., Hähner A., Ultsch A., Hill K., Hummel T. (2015). A brain-lesion pattern based algorithm for the diagnosis of posttraumatic olfactory loss. Rhinology.

[106] Morse J.A.-O.X., Shilts M.H., Ely K.A., Li P., Sheng Q., Huang L.C., Wannemuehler T.J., Chowdhury N.I., Chandra R.K., Das S.R. (2019). Patterns of olfactory dysfunction in chronic rhinosinusitis identified by hierarchical cluster analysis and machine learning algorithms. Int. Forum Allergy Rhinol..

[107] Walker A., Surda P. (2019). Unsupervised Learning Techniques for the Investigation of Chronic Rhinosinusitis. Ann. Otol. Rhinol. Laryngol..

[108] Ward Jr J.H. (1963). Hierarchical grouping to optimize an objective function. J. Am. Stat. Assoc..

[109] MacQueen J. Some methods for classification and analysis of multivariate observations. Proceedings of the Fifth Berkeley Symposium on Mathematical Statistics and Probability.

[110] Ultsch A., Lötsch J. (2017). Machine-learned cluster identification in high-dimensional data. J. Biomed. Inform..

[111] Ester M., Kriegel H.-P., Sander J.o., Xu X. A Density-Based Algorithm for Discovering Clusters in Large Spatial Databases with Noise. Proceedings of the Second International Conference on Knowledge Discovery and Data Mining.

[112] Yaohui L., Zhengming M., Fang Y. (2017). Adaptive density peak clustering based on K-nearest neighbors with aggregating strategy. Knowl. Based Syst..

[113] Lötsch J., Hummel T. (2019). A machine-learned analysis suggests non-redundant diagnostic information in olfactory subtests. IBRO Rep..

[114] Ramakrishnan V.R., Arbet J., Mace J.C., Suresh K., Shintani Smith S., Soler Z.M., Smith T.L. (2021). Predicting Olfactory Loss In Chronic Rhinosinusitis Using Machine Learning. Chem. Senses.

